# Zebrafish Reveals Genotype‐Specific Differences in Betanodavirus Infection and Host Responses

**DOI:** 10.1155/tbed/8111758

**Published:** 2026-10-30

**Authors:** Patricia Moreno, Daniel Álvarez-Torres, Manon Mehraz, Candela Ravina, Maxence Frétaud, Manuel Marí-Beffa, M. Carmen Alonso, Esther García-Rosado, Christelle Langevin, Julia Béjar

**Affiliations:** ^1^ Facultad de Ciencias Departamento de Microbiología Instituto de Biotecnología y Desarrollo Azul, IBYDA Universidad de Málaga Málaga 29071 Spain uma.es; ^2^ Facultad de Ciencias Departamento de Biología Celular Genética y Fisiología Instituto de Biotecnología y Desarrollo Azul, IBYDA Universidad de Málaga Málaga 29071 Spain uma.es; ^3^ Infectiologie Expérimentale des Rongeurs et des Poissons INRAE Université Paris-Saclay Jouy-en-Josas France universite-paris-saclay.fr; ^4^ INRAE UVSQ Virologie et Immunologie Moléculaires Université Paris-Saclay Jouy-en-Josas France universite-paris-saclay.fr

**Keywords:** innate immune response, RGNNV, RGNNV/SJNNV, SJNNV, zebrafish

## Abstract

Fish viral encephalopathy and retinopathy (VER) is a significant infectious disease caused by the nervous necrosis virus (NNV), which affects cultured and wild species. Four NNV genotypes with different virulence in susceptible species have been identified: red‐spotted grouper NNV (RGNNV), striped jack NNV (SJNNV), barfin flounder NNV (BFNNV), and tiger puffer NNV (TPNNV), in addition of reassortment between RGNNV and SJNNV. In light of this threat, zebrafish emerged as a promising model to further characterize these pathogens and identify the cellular and molecular mechanisms responsible for susceptibility/resistance phenotypes. Thus, the aim of the present study was to compare the susceptibility of zebrafish to RGNNV, SJNNV, and RGNNV/SJNNV infection and to characterize the pathogenesis of these isolates in terms of virulence and early innate host immune response. Disease symptoms were recorded in all groups of intracranially infected larvae, and quantification of viral genome evidenced that the three isolates replicate in zebrafish larvae. The highest mortality and proportion of larvae showing disease symptoms were registered in the RGNNV group, where the highest viral replication level was also recorded. Thus, RGNNV seems to be the most virulent isolate for zebrafish larvae. Interestingly, transcriptional patterns of IFN I response and apoptosis genes were similar for RGNNV‐ and RGNNV/SJNNV‐inoculated larvae, being interferon regulatory factor 3, *irf3*, and receptor transporter protein 3, *rtp3*, the most induced genes, whereas a much lower gene upregulation was recorded in SJNNV‐inoculated larvae. RGNNV infection also induced stronger inflammatory responses, including neutrophil recruitment. RGNNV infection of zebrafish larvae by immersion lead to a significant increase of viral genome; however, the infection was quickly controlled. Infection of juvenile zebrafish with RGNNV by intramuscular injection lead to virus migration to the central nervous system; however, no viral RNA replication was recorded. The potential of zebrafish to study NNV‐host interplay prompts further studies into the underlying mechanisms of these observations, which could contribute to design effective strategies to protect farmed fish susceptible to NNV infections.

## 1. Introduction

Viral nervous necrosis (VNN), also known as viral encephalopathy and retinopathy (VER), is responsible for important economic losses in aquaculture facilities, affecting the main fish species cultured in Mediterranean countries. Affected fish display altered swimming behavior and important histopathological lesions, consisting of vacuolation and necrosis of cells in the brain, spinal cord, and retina, which are the target organs for viral replication [1]. The causative agent of this disease is the nervous necrosis virus (NNV, *Nodaviridae* family, *Betanodavirus* genus), a small, non‐enveloped virus, with a genome composed of two positive‐sense RNA segments: RNA1, which encodes the RNA‐dependent RNA polymerase; and RNA2, encoding the capsid protein (CP). In addition, a sub‐genomic segment, RNA3, is synthesized during RNA replication from the RNA1 3^′^‐end. RNA3 comprises two open reading frames that encode two non‐structural proteins, B1 and B2.

On the basis of the T4 sequence, within the RNA2 segment, four betanodavirus genotypes have been established [2]: red‐spotted grouper NNV (RGNNV), striped jack NNV (SJNNV), barfin flounder NNV (BFNNV), and tiger puffer NNV (TPNNV); although only RGNNV and SJNNV have been detected in the Mediterranean area to date. Moreover, RGNNV–SJNNV reassortant viruses, presenting both segment combinations, SJNNV/RGNNV and RGNNV/SJNNV (RNA1/RNA2), have been isolated or detected from several fish species [3–6]. A large number of NNV‐susceptible fish species have been reported, including economically relevant species, such as European sea bass (*Dicentrarchus labrax*), gilthead sea bream (*Sparus aurata*), Senegalese sole (*Solea senegalensis*), and grouper (*Epinephelus* spp.) [3, 4, 6–8]; however, not all genotypes are equally virulent to each specific host species. Thus, RGNNV is highly virulent to sea bass [8–10], in contrast to RGNNV/SJNNV and SJNNV isolates, which cause very low mortality in this fish species [8, 10, 11]. On the contrary, SJNNV and RGNNV/SJNNV isolates are highly virulent to Senegalese sole and sea bream [3, 4, 6, 12]. This variation in disease severity induced by different betanodaviruses across host species highlights the importance of performing comparative analyses of their virulence, mechanisms of infection, and modulation of host responses.

The general advantages of using zebrafish larvae (*Danio rerio*) as a model for in vivo investigations of viral diseases are widely known [13], particularly to study antiviral innate immune responses before the development of adaptive immune system, which takes place at 4–6 weeks post fertilization [14]. Thus, it has been used to model infectious processes and identify virus entry and dissemination, as well as to unravel host immune responses by combining advanced imaging methods and molecular biology. The development of infection models includes human viruses such as noroviruses, influenza A virus, chikungunya virus, zika virus, dengue virus, herpes simplex virus type 1, Sindbis virus, human cytomegalovirus, and hepatitis B and C viruses, as well as severe acute respiratory syndrome coronavirus 2 (reviewed by [15, 16]). Furthermore, zebrafish are also susceptible to a wide range of DNA and RNA fish viruses, including hematopoietic necrosis virus [17, 18], viral hemorrhagic septicemia virus [19], infectious pancreatic necrosis virus [20], snakehead rhabdovirus [21], infectious spleen and kidney necrosis virus [22, 23], European sheatfish virus [24], and spring viremia of carp virus [25–29].

In addition, the small size and transparency of embryos and larvae are of particular interest for drug screening, allowing to assess drug effects on organ development and larval behavior [30–32] as well as for the identification of novel drug targets and the evaluation of immunomodulators or antiviral compounds [15, 16, 33]. The lack of licensed antiviral treatments to be used in fish farming significantly hampers the control of disease outbreaks. Consequently, the development and implementation of effective antiviral strategies is a key priority and a major challenge for the aquaculture sector. In this context, zebrafish is a valuable platform capable of assessing and comparing the antiviral efficacy of candidate compounds, enabling the evaluation and optimization of their in vivo application [34, 35].

Establishment of a zebrafish model to study NNV infections has been reported using the RGNNV genotype [36, 37], leading to a productive infection, activation of IFN responses, and mortality rates dependent on the viral dose, administration route, and stage of fish development. Moreover, the model was pertinent to show the anti‐NNV effect of ribavirin [38]. Interestingly, RGNNV infection of zebrafish larvae recapitulates RGNNV pathology in sea bass, marked by histopathological lesions in the central nervous system and altered swimming behavior [36, 37, 39, 40]. However, other NNV genotypes have not been tested in zebrafish to date.

The current study used zebrafish as a model to investigate the mechanisms involved in the resistance‐susceptibility pattern to different NNV genotypes. Thus, the aim of the present study was to compare the susceptibility of zebrafish to RGNNV, SJNNV, and RGNNV/SJNNV isolates and to characterize the infection caused by these isolates in terms of virulence and early host immune response combining fish imaging and molecular biology.

## 2. Material and Methods

### 2.1. Virus Propagation

E11 cells [41] were grown on 25 cm^2^ flasks (Nunc, ThermoFisher) at 25°C with Leibovitz medium (L15, Gibco) supplemented with 5% foetal bovine serum (FBS, Gibco), 100 units/mL penicillin, and 10 mg/mL streptomycin (Sigma).

The isolates SpDl_IAusc965.09 (Dl956, RGNNV), obtained from sea bass [42]; SJ93Nag, a reference SJNNV strain obtained from striped jack (*Pseudocaranx dentex*), as well as an RGNNV/SJNNV isolate, obtained from sea bream, were propagated on E11 cells grown with L15 medium supplemented with 2% FBS, 100 units/mL penicillin, and 10 mg/mL streptomycin. Inoculated cells were incubated at 25°C (RGNNV and RGNNV/SJNNV) or at 22°C (SJNNV) until extensive cytopathic effects (CPEs) were observed. The viral suspensions obtained were titrated following the 50% tissue culture infective dose method (TCID_50_) [43] and stored at −80°C until used.

### 2.2. NNV Infection of Zebrafish Larvae by Intracranial Injection

Wild‐type (WT) AB‐strain zebrafish larvae at 3 days post‐fertilization (dpf) were distributed in five groups: (i) RGNNV‐infected; (ii) SJNNV‐infected; (iii) RGNNV/SJNNV‐infected; (iv) L15‐injected maintained at 22°C (control group, C22); and (v) L15‐injected maintained at 25°C (control group, C25). A total of 80 larvae were injected per group, 40 larvae (distributed into four wells of a six‐well plate, 10 larvae/well) were used to determine mortality and record symptoms, and 40 larvae (distributed into six wells of a six‐well plate, 6–7 larvae/well) for sampling. Larvae were anesthetized with tricaine (0.005% MS‐222, Sigma) and intracranially injected (5 nL) with NNV isolates (50 TCID_50_/fish) or L15 medium (control groups, C, mocked‐infected larvae). Each infected group was maintained at the optimal temperature for viral replication, which is 22°C for SJNNV and 25°C for both RGNNV and RGNNV/SJNNV [44]; consequently, a control group at each temperature was set up. Injections were performed with filamented glass microcapillaries (GC100F‐15, Phymep) using a mechanical micromanipulator (M‐152, Narishige) and a Femtojet microinjector (Eppendorf). To minimize potential physiological alterations due to abrupt temperature changes, larvae were maintained in 2 mL of E3 medium at 22°C (SJNNV and C22) or 25°C (RGNNV, RGNNV/SJNNV, and C25) before and after injection. Following infection, larvae were daily monitored for clinical signs of disease and mortality for 4 days. At 1 and 4 days post‐inoculation (dpi), three replicates, consisting of pools of 6 larvae from different wells, were sampled per group and stored in the Trizol reagent (Sigma–Aldrich) at −80°C until used.

### 2.3. Quantification of Viral RNA and Immune Gene Transcription

RNA extraction and cDNA synthesis were conducted according to Moreno et al. [42]. Larvae were homogenized (10%, w/v) in the TRI reagent to extract total RNA following commercial instructions. After treatment with DNase I Recombinant from bovine pancreas, cDNA was synthesized using 1 μg of RNA and the Transcriptor First Strand cDNA Synthesis Kit according to the manufacturer’s guidelines. cDNA was quantified with a NanoDrop‐1000 system and maintained at −20°C until used.

The viral RNA2 segment was quantified by absolute qPCR performed according to Moreno et al. [45]. Briefly, serial dilutions of the *pJET* vector (Thermo Scientific) containing the complete RNA2 sequence were used to generate the standard curve. Amplifications were conducted with a LightCycler 96 Thermocycler and 20 µL mixtures containing 2 µL of cDNA obtained from 50 ng of RNA, 10 µL of Fast Start Essential DNA Green Master mix (Roche), and primers shown in Table S1 (0.5 µM, final concentration). The amplification profile was 10 min at 95°C, followed by 45 cycles of 95°C for 10 s, 60°C for 10 s, and 72°C for 10 s, and a final step of 95°C for 10 s.

To analyze the immune response, a transcriptomic analysis focused on genes involved in the type I interferon (IFN I) response (myxovirus influenza resistance A, *mxa*, ISG15 ubiquitin‐like modifier, *isg15*, receptor transporter protein 3, *rtp3* interferon regulatory factor 3, and *irf3*), apoptosis (caspase 8, *casp8*, caspase 9, *casp9*, *cathepsin La*, and *ctsl1a*), and inflammation (tumor necrosis factor a, *tnfa*, interleukin 1 beta, *il1b*, interleukin 6, *il6*, interleukin 8, *il8*) was conducted by qPCR, using a LightCycler 96 Thermocycler, and 20 µL mixtures containing 10 µL of the Fast Start Essential DNA Green Master Mix, 1 µL of each primer (0.5 µM, final concentration) (Table S1) and 2 µL of cDNA (generated from 50 ng of RNA). The amplification profile was as follows: 95°C for 10 min, followed by 40 cycles of 95°C for 10 s, 60°C for 10 s, 72°C for 10 s, and a final step of 95°C for 10 s. Fold change values (FC) were calculated by the 2^−∆∆Ct^ method, using actin beta 1, *actb1*, as the endogenous gene (Table S1), and a pool of six mock‐infected larvae (control group) as the calibrator sample.

### 2.4. Fluorescence Imaging and Image Analysis

Fluorescence imaging of *Tg*(*mpx:GFP*)*i114* transgenic larvae [46] was carried out to observe the distribution of neutrophils in whole control and infected larvae. *Tg*(*mpx:GFP*)*i114* larvae at 3 dpf were distributed in five groups of 10 larvae each, using six‐well plates: (i) RGNNV‐infected; (ii) SJNNV‐infected; (iii) RGNNV/SJNNV‐infected; (iv) L15 injected maintained at 22°C (control group C22); and (v) L15 injected maintained at 25°C (control group C25). Larvae were anesthetized, intracranially injected with NNV isolates or L15 medium, and maintained as described above. Acquisitions of GFP fluorescence and transmitted light were performed at 1 and 4 dpi with an MZ10F fluorescent stereomicroscope equipped with an external light source (Leica EL6000), a PLAN APO 1× objective, and a MC170HD camera (Leica Microsystems).

Images were processed with Fiji (https://imagej.net/software/fiji/). The automatic quantitation of neutrophil fluorescence signals was carried out with a homemade Fiji macro. First, regions of interest (ROI) were designed for the head, head kidney, and posterior part of the body (trunk); an auto‐threshold was then applied, followed by conversion of the images into mask. Finally, mean gray values were measured for each ROI and normalized to the total fluorescence signal detected across the entire larvae.

### 2.5. RGNNV Infection of Zebrafish Larvae by Immersion

Three groups of WT zebrafish larvae (AB strain, 3 dpf, *n* = 12/group), corresponding to three independent replicates, were maintained at 25°C in E3 medium containing RGNNV (5 × 10^6^ TCID_50_/mL). In addition, three control groups consisting of larvae maintained in E3 medium with L15 were also established (AB strain, 3 dpf, *n* = 12/group). At 0, 2, and 6 dpi, three larvae per group were euthanized by anesthetic overdose, pooled, and homogenized in Trizol Reagent to obtain total RNA and cDNA as described above. Thus, three pools/time point were analyzed from both infected and control groups. Viral RNA2 segment and transcription of genes involved in IFN I response (*isg15*, *rtp3*, and *irf3*), apoptosis (*casp8*, *ctsl1a*), and inflammation (*il1b* and *tnfa*) were quantified by qPCR as described above.

### 2.6. RGNNV Infection of Juvenile Zebrafish by Intramuscular Injection

Three groups (*n* = 12/group) of juvenile zebrafish WT (AB strain, 60 dpf) were anesthetized and intramuscularly injected with an RGNNV inoculum (10^5^ TCID_50_/g of fish). In addition, three control groups (*n* = 12) were injected with L15 medium (mock‐infected group). At 1, 3, and 6 dpi, three fish per group were euthanized by anesthetic overdose, and heads (since brain and retina are NNV target organs) were collected, pooled, and stored in Trizol Reagent at −80°C for the subsequent total RNA extraction and cDNA synthesis, as previously described. Thus, three pools/time point were analyzed from both infected and control groups. Viral RNA2 segment and transcription of genes involved in IFN I response (*isg15*, *rtp3*, and *irf3*), apoptosis (*casp8* and *ctsl1a*), and inflammation (*il1b* and *tnfa*) were quantified by qPCR as described above.

### 2.7. Statistical Analyses

Data derived from viral replication and transcriptomic analyses were statistically analyzed using GraphPad Prism 6 software (GraphPad Software, Inc., La Jolla, USA). The normality distribution of the data was assessed using the Shapiro–Wilk test. The one‐way ANOVA test, followed by the Tukey’s multiple comparison test as the post‐hoc analysis, was used. Statistical analyses for pixel count were performed using the non‐parametric Mann–Whitney test on GraphPad Prism 8 software. Values of *p* < 0.05 were considered significant.

### 2.8. Ethical Statement

All larvae and juveniles were handled in strict accordance with good animal practice, as defined by the European Union guidelines for the handling of laboratory animals (http://ec.europa.eu/environment/chemicals/lab_animals/home_en.htm). All challenges were performed in accordance with the European Directive 2010/2063/UE regarding animal experiments. The protocols were evaluated and approved by the ethical committee COMETHEA n°45 of the INRAE Center in Jouy en Josas‐Antony, and the Direction of the Veterinary Services of Versailles, France (building agreement number D78‐322‐720), as well as the ethical committee of the Universidad de Málaga and the Regional Government (Ref. no. 04/05/2021/072).

## 3. Results

### 3.1. Susceptibility of Zebrafish Larvae to RGNNV, RGNNV/SJNNV, and SJNNV

The susceptibility of zebrafish larvae to each viral isolate was first assessed through daily monitoring of intracranially‐injected larvae, in order to detect the onset of clinical signs and to record mortality rates (Figure 1). Larvae intracranially infected with either RGNNV, RGNNV/SJNNV, or SJNNV showed edemas from 1 dpi, which were not detected in any of the control groups (22 and 25°C). This phenotype is particularly pronounced in the RGNNV‐inoculated group, affecting ~40% of individuals from 2 dpi, after a significant increase between 1 and 2 dpi and reaching a plateau thereafter. In contrast, the proportion of larvae displaying viral‐induced edema does not exceed 3% (one individual) and 10% for RGNNV/SJNNV and SJNNV, respectively, without evolution during the time course of the infection. The reassortant RGNNV/SJNNV also induced body curvature in 3% of larvae (one individual) from 3 dpi onwards. Although clinical signs were observed across all infected groups, mortality was restricted to the RGNNV‐infected group (increasing between 1 and 4 dpi) and, to a lesser extent, to the SJNNV group, reaching cumulative mortality rates of 14% and 6%, respectively. Initial mortality in the C22 control group at 1 dpi was attributed to handling or injection trauma.

**Figure 1:**
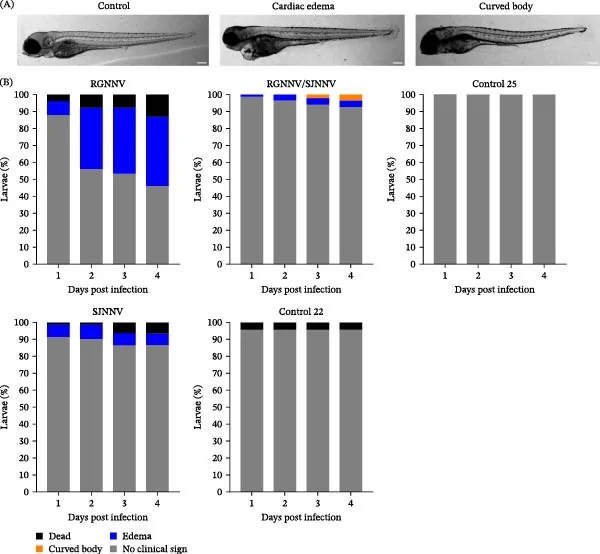
Disease symptoms and mortality of zebrafish larvae intracranially infected with RGNNV, RGNNV/SJNNV, or SJNNV. (A) Disease symptoms. Scale bar: 200 µm. (B) Percentage of larvae showing each clinical sign.

The replication of each viral isolate was examined by quantifying the viral genome. The results shown in Figure 2 evidenced a significant increase in the number of RNA2 copies from 0 to 4 dpi (*p* < 0.05) in all groups, indicating that the three NNV isolates replicate in zebrafish larvae (Figure 2). However, some differences were recorded depending on the viral isolate; thus, the number of RNA2 copies increased continuously (from 0 to 4 dpi) in RGNNV‐ and RGNNV/SJNNV‐inoculated larvae, whereas no significant increase was recorded from 1 to 4 dpi in the SJNNV group (*p* < 0.05). In addition, the maximum mean copy number was recorded in the RGNNV‐inoculated group (*p* < 0.05), indicating a higher level of replication of this isolate in zebrafish larvae, compared with SJNNV and RGNNV/SJNNV replication.

**Figure 2:**
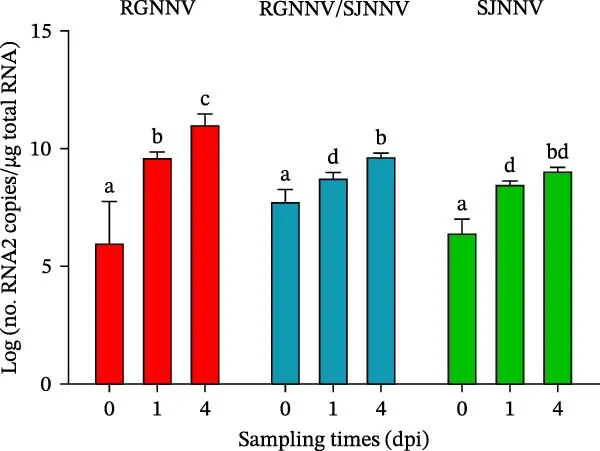
RNA2 quantification in zebrafish larvae intracranially infected with RGNNV (red), RGNNV/SJNNV (blue), or SJNNV (green). Data were analyzed with the two‐way ANOVA test. Different letters denote significant differences. Values of *p* < 0.05 were considered significant. Results are mean ± SD.

### 3.2. Immune Response of Intracranially Infected Zebrafish Larvae

To evaluate immune responses across infected groups and their association with the pathophysiology of the disease (symptoms and mortality), we conducted transcriptional analyses of larvae collected at 1 and 4 dpi (Figure 3). A significant upregulation of IFN I‐stimulated genes (*mxa*, *isg15*, *rtp3*, and *irf3*) was recorded in RGNNV‐ and RGNNV/SJNNV‐inoculated larvae at both sampling times, as well as in the SJNNV group, although to a lower level and only at 4 dpi (Figure 3A). The apoptosis‐associated markers *casp8* and *casp9* were significantly upregulated only at 4 dpi in all infected groups. In addition, significant upregulation of *ctsl1a* at 4 dpi was also induced by the RGNNV infection (Figure 3B). Inflammatory responses were assessed by analyzing the transcription of *il1b*, *tnfa*, and *il6* cytokines, in addition to *il8*, shown to be involved in neutrophil recruitment and inflammation resolution [47] (Figure 3C). Inoculation of RGNNV led to a significant upregulation of *il1b, tnfa*, and *il8* only at 4 dpi. In the RGNNV/SJNNV group, *tnfa* and *il6* were induced at 4 dpi, whereas in the SJNNV‐inoculated group, *tnfa* was induced at 1 and 4 dpi, whereas *il6* was induced only at 4 dpi.

**Figure 3:**
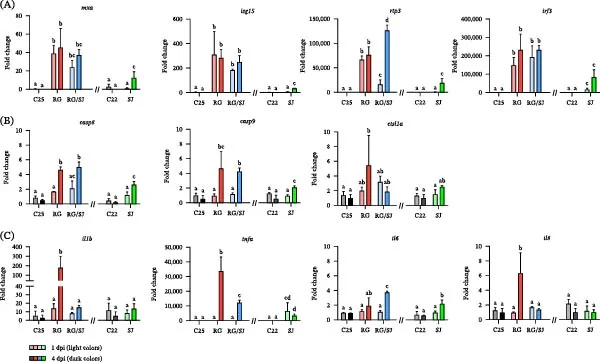
Transcription quantification of genes related to (A) IFN I system; (B) apoptosis; and (C) inflammatory response in zebrafish larvae inoculated by intracranial injection with RGNNV (RG, red), RGNNV/SJNNV (RG/SJ, blue), and SJNNV (SJ, green) viruses. Data were analyzed with the two‐way ANOVA test. Different letters denote significant differences. Values of *p* < 0.05 were considered significant. Results are mean ± standard deviation (SD).

To further characterize the fish immune response at the cellular level, we carried out infection of transgenic larvae expressing fluorescent GFP protein in neutrophils. Fluorescence imaging at cellular resolution enabled the detection of neutrophils at the whole‐body level to observe their distribution in the steady state and in NNV‐infected conditions. In naïve conditions, neutrophils were mainly localized in the caudal hematopoietic tissue as well as in the patrolling sparse cells detected throughout the whole body (Figure 4A). Quantitation of GFP intensity was conducted to measure neutrophil recruitment within ROI and was normalized to the total GFP intensity of the whole larvae to account for interindividual differences in cell number. RGNNV and, to a lesser extent, RGNNV/SJNNV inoculations triggered significant neutrophil recruitment in the head (*p* value 0.001 and 0.035, respectively) and head kidney (*p* value 0.01 and 0.017) at 1 dpi (Figure 4B). These differences were not observed at 4 dpi. In contrast, SJNNV inoculation did not induce neutrophil redistribution to the head or head kidney and exhibited a distribution profile comparable to that of the control group (C22) at the analyzed time points (Figure 4B).

**Figure 4:**
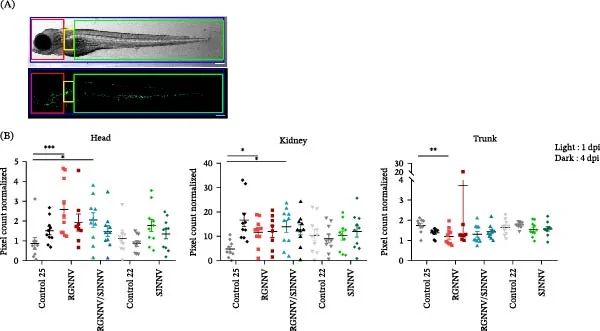
Neutrophil recruitment in *Tg*(*mpx:GFP*) zebrafish larvae intracranially infected. (A) Representatives ROI for the pixel count (blue: body, red: head, yellow: kidney, and green: trunk). Top panel: brightfield *Tg*(*mpx:GFP*) 3 dpf control larvae; bottom panel: fluorescent *Tg*(*mpx:GFP*) 3 dpf control larvae. The pixel counting was performed on the fluorescence images. Scale bar: 200 µm. (B) pixel count normalized for each ROI (head, kidney, and trunk) in fish infected with RGNNV (red), RGNNV/SJNNV (blue), or SJNNV (green). Larvae injected with L15 and incubated at 25 or 22°C were used as controls (C25, black/dark gray; or C22, gray/light gray). Data were analyzed with the Mann–Whitney test. Asterisks denote significant differences ( ^∗^ *p* < 0.05,  ^∗∗^ *p* < 0.01,  ^∗∗∗^ *p*  < 0.001).

Altogether, these results indicate that RGNNV and RGNNV/SJNNV infections triggered similar IFN I and apoptotic responses, whereas SJNNV elicited a weaker activation of these pathways. By combining fluorescence imaging and transcriptional analyses, we further demonstrate that the RGNNV isolate induced stronger inflammatory responses, including neutrophil recruitment.

### 3.3. Immersion Infection of Zebrafish Larvae With RGNNV

The waterborne infection model, based on fish immersion in viral suspensions, is less invasive and stressful than viral administration by injection and mimics natural routes of virus entry in target species. Having established the higher virulence of RGNNV in zebrafish larvae intracranially infected, we tested the waterborne infection with this isolate. Zebrafish exposure to RGNNV by immersion resulted in a slight but significant increase of the viral RNA2 copy number from 0 to 2 dpi (Figure 5A), although neither mortality nor disease symptoms were observed. The transcription analysis of immune‐related genes in infected larvae showed a clear upregulation of genes belonging to the type‐I IFN signaling pathway (Figure 5B) and apoptotic response (Figure 5C) at 6 dpi. However, in contrast to the results derived from the intracranial injection with the same isolate (Figure 3C), proinflammatory genes were not significantly induced at any sampling time tested (Figure 5D).

**Figure 5:**
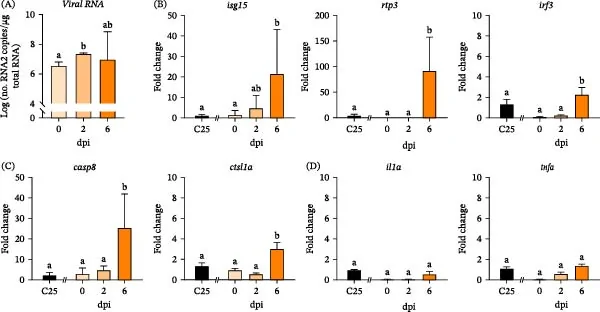
Quantification of (A) RGNNV RNA2, (B) IFN I system genes, (C) apoptosis genes, and (D) inflammatory response genes in zebrafish larvae infected with RGNNV by immersion. Data were analyzed with the two‐way ANOVA test. Different letters denote significant differences between the C25 control group and the experimental group. Values of *p* < 0.05 were considered significant. Results are mean ± standard deviation (SD).

### 3.4. Intramuscular Infection of Zebrafish Juveniles With RGNNV

In order to complete the characterization of betanodavirus infection in zebrafish, the RGNNV infection was examined in juveniles. Upon intramuscular RGNNV inoculation, the standard method in fish betanodavirus experimental infections due to its high efficiency [48], neither mortality nor disease symptoms were observed, at least up to 6 dpi. Furthermore, the RNA2 copy number in the head did not significantly increase over time, suggesting the absence of viral replication (Figure 6A).

**Figure 6:**
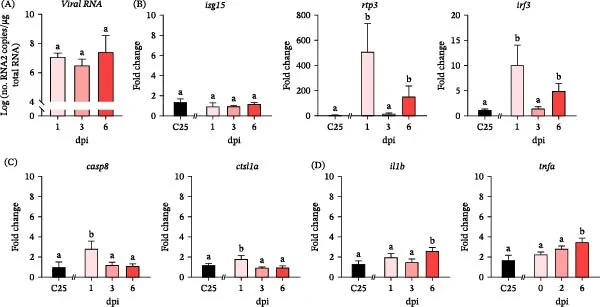
Quantification of (A) RGNNV RNA2, (B) IFN I system genes, (C) apoptosis genes, and (D) inflammatory response genes in juvenile zebrafish infected with RGNNV by intramuscular injection. Data were analyzed with the two‐way ANOVA test. Different letters denote significant differences between the C25 control group and the experimental group. Values of *p* < 0.05 were considered significant. Results are mean ± standard deviation (SD).

To evaluate the putative relationship between the lack of viral replication and the host immune response, the RGNNV‐induced modulation of immune‐related genes was examined. Both *rtp3* and *irf3* displayed a marked induction at 1 dpi before declining and rising to a second peak at 6 dpi (Figure 6B). In parallel, a significant but transient induction of *casp8* and *ctsl1a* was detected at 1 dpi (Figure 6C), whereas a significant upregulation of inflammatory genes (*tnfa* and *il1b*) was observed at 6 dpi (Figure 6D).

## 4. Discussion

VER, caused by NNV, are one of the main threats for the fish farming industry, causing high mortality rates in early developmental stages [49]. Numerous fish species worldwide are susceptible to betanodaviruses, including zebrafish, which has been proven to be susceptible to the RGNNV genotype [36–38, 40]. However, a given fish species may not be susceptible to all NNV genotypes [50]. Thus, the objective of the present study was to determine zebrafish susceptibility to different betanodavirus genotypes and to characterize the early innate immune response triggered by each virus in this model species since variations in initial host immune response may underlie susceptibility differences.

The susceptibility of zebrafish to NNV isolates was assessed in larvae inoculated by an intracranial injection. The three isolates tested replicated and generated disease symptoms, which indicates that zebrafish larvae are susceptible to RGNNV, SJNNV, and RGNNV/SJNNV isolates, even though SJNNV replication seems to be controlled at 4 dpi, and the percentage of symptomatic RGNNV/SJNNV‐infected larvae was low. Interestingly, mortality of SJNNV‐inoculated larvae occurred when viral replication seemed to stabilize, suggesting that the damage caused during the early viral replication phase continues progressing and eventually leads to death. It is tempting to associate this damage with the very early (1 dpi) and strong (FC > 5000) *tnfa* induction recorded only in SJNNV‐infected larvae at this time point. To the best of our knowledge, this is the first study showing zebrafish larvae susceptibility to RGNNV/SJNNV and SJNNV. Regarding the severity of the disease, the highest mortality and proportion of larvae showing disease symptoms were registered in the RGNNV group (Figure 1). Moreover, RGNNV was the viral isolate with the highest replication level (Figure 2). Thus, RGNNV seems to be the most virulent isolate for zebrafish larvae. Previous RGNNV infections yielded different mortality rates depending on the larval age and the inoculation route. In the current study, RGNNV caused 2.4% mortality at 4 dpi, whereas Lama et al. [37] registered 44% mortality at 10 dpi using the same inoculation route and larval age. We did not observe stabilization of mortality at 4 dpi (Figure 1), and therefore, a higher level of mortality would have been expected at 10 dpi. Since this study focused on the early infection dynamics rather than on the determination of ultimate survival, an early end‐point of 4 dpi was predefined. Therefore, longer‐term effects could not be determined and must be studied in further experiments with extended endpoints. Regarding clinical signs, Morick et al. [40] reported histopathological lesions in the brain and retina, whereas Lama et al. [37] described altered swimming behavior of infected larvae; however, neither curved body nor edema in NNV‐infected larvae had been reported before. Cardiac edema has been widely associated to toxicity [51] or ciliopathies [52] in zebrafish larvae, whereas curved body has been associated to anomalies in the central canal, Reissner’s fiber, and subcommisural organ secretory activity [53], increased mechanical loads induced by swimming activity [54], ciliopathies [52], inflammation, oxidative stress, cerebrospinal flow affections and neurodegeneration, or *Pseudoloma neurophilia*, but not *Staphylococcus aureus*, infection [55]. This constellation of potential causes has been recently unified in the activity of a single ciliated proprioceptive organ in the neural tube ependyma that may act as a mechanoreception system of the cerebrospinal fluid flow (CFF) [55]. This organ may transduce CFF into muscle contraction and postural control by projecting axons that activate inter‐ and motor neurons ipsilaterally [55]. Our study adds a new body curvature‐inducing factor, the intracranial infections of the reassortant RGNNV/SJNNV. The presence of RGNNV/SJNNV in the cerebrospinal fluid may affect ciliated ependymal organs in the neural tube that includes this proprioceptive organ. Nevertheless, this effect has occurred at a low frequency.

Several studies have identified the IFN I system, apoptosis, and inflammation as key pathways in controlling the onset and severity of the VER disease in different economically important fish species [56–58]. Thus, we compared the induction of these pathways to identify genotype‐specific host responses that contribute to disclose key aspects of betanodavirus‐host interplay responsible for the outcome of infections. RGNNV triggered an early IFN I response (*mxa*, *isg15*, *rtp3*, and *irf3*) followed by the upregulation of most of the apoptotic and proinflammatory cytokines tested (*casp8*, *casp9*, *tnfa*, *il1b*, and *il8*) (Figure 3), which is the expression pattern recorded in other susceptible fish species showing VER disease, such as European sea bass [58], Senegalese sole [57], or grouper [56], as well as in zebrafish [37].

Interestingly, the induction patterns of IFN I response and apoptosis genes in RGNNV‐ and RGNNV/SJNNV‐inoculated larvae were similar; only *rtp3* showed a delayed but stronger response at 4 dpi in the RGNNV/SJNNV group. The strong *irf3* response in RGNNV‐ and RGNNV/SJNNV‐inoculated larvae (FC > 200,000) might be related with the similar induction of apoptosis‐related genes in these two groups of larvae since, in addition to being involved in type I *ifn* transcription induction upon viral infections, IRF3 also stimulates apoptosis [59]. Regarding the prominent response of *rtp*3, our results agree with previous studies that demonstrated a strong correlation between the induction of *irf3* and *rtp3* transcription [60]. Actually, we recorded this correlation also in the immersion and intramuscular injection assays (Figures 5B and 6B). Moreover, a strong and quick induction of *rtp3* has been reported after betanodavirus infection in several fish species [58, 61, 62]; and European sea bass *rtp3* genes are involved in several processes related with betanodavirus pathogenesis [63]; therefore, the role of this gene in NNV–zebrafish interaction could also be essential, as it has also been suggested for Asian sea bass [64]. In contrast, a much lower upregulation of IFN I response and apoptosis genes was recorded in SJNNV‐inoculated larvae.

Regarding the inflammatory response, *tnfa* and *il6* were slightly induced in SJNNV‐infected larvae (Figure 3C). Interestingly, RGNNV/SJNNV‐inoculated larvae also showed a low induction of the same two inflammatory response genes (*tnfa* and *il6*) (Figure 3C), whereas RGNNV infection triggered a higher induction of proinflammatory genes (*il1b*, *tnfa*, and *il8*) at 4 dpi (Figure 3C), similarly to the results obtained by Lama et al. [37]. The low induction of inflammatory genes triggered by SJNNV and RGNNV/SJNNV infection could be involved in the low virulence of these two isolates since a mild inflammatory response has been related to the lack of mortality and symptoms of the VER disease [58, 65]. Consistent with this idea, neutrophil dynamic analyses, used as an indicator of early inflammatory response, revealed that neutrophil migration to the head was higher in RGNNV‐infected than in RGNNV/SJNNV‐infected larvae at the beginning of the infection (1 dpi) (Figure 4B). This early recruitment of neutrophils agrees with the well‐established role of these cells as the first immune cell population arriving at infection sites [66], as well as with the results obtained by Lama et al. [37]. Furthermore, in a zebrafish scoliosis model (*sspo* zebrafish mutants), both macrophage migration and *tnfa* over‐expression have been associated to neuroinflammation in the curved body [67]. These results could explain the late expression of *tnfa* and the increase of trunk macrophage migration observed in RGNNV/SJNNV‐inoculated larvae.

Thus, the induction of IFN I‐ and apoptosis‐related genes and the early neutrophil recruitment were similar in RGNNV and RGNNV/SJNNV‐inoculated larvae, whereas the induction of inflammation‐related genes at 4 dpi was similar in RGNNV/SJNNV and SJNNV‐inoculated larvae. Therefore, differences in the virulence of the three viruses could be related to the inflammatory response, driven initially by neutrophil recruitment and later on by the induction of proinflammatory genes. Actually, Lama et al. [37] reported that RGNNV infection in zebrafish larvae triggered both strong antiviral response and significant upregulation of pro‐inflammatory cytokines, and similar results were obtained in Atlantic halibut, *Hippoglossus hippoglossus* [68], and European sea bass [69]. In contrast, gilthead sea bream, which is considered resistant to RGNNV infection, also mounted a robust antiviral response while displaying a much more controlled inflammatory profile that was slightly different after infection with an RGNNV/SJNNV isolate, which causes high mortality at the larval stage in this fish species [4, 60, 70]. Furthermore, it is well established that the balance between immune control and immune evasion or overreaction, that is, excessive inflammation, is key during viral infections and may be responsible for the severity of viral diseases [71]. Therefore, studying the mechanisms underlying this balance is crucial to understand the NNV‐host interplay.

Apart from the host immune response, each viral NNV genotype displays specific virulence features. Specifically, SJNNV differs from RGNNV and RGNNV/SJNNV in the RNA1 segment, which encodes two proteins involved in the antagonistic activity and host cell death, B1 and B2 [72–74]. Therefore, differences in the antagonistic capacity of SJNNV and both RGNNV and RGNNV/SJNNV isolates could account for the distinct levels of IFN I‐response induction observed [75]. Furthermore, the similar apoptotic response recorded in RGNNV‐ and RGNNV/SJNNV‐inoculated larvae also supports the role of B1 and B2 in the transcriptional response triggered by betanodaviruses. On the other hand, RGNNV differs from RGNNV/SJNNV and SJNNV isolates in the RNA2 segment that encodes the CP protein. Several studies have demonstrated that two amino acids located in the protruding domain of the C‐terminus region are important determinants of RGNNV virulence to Senegalese sole, turbot (*Scophthalmus maximus*), and European sea bass [8, 42, 58, 76]. Since RGNNV is the most virulent isolate in zebrafish, the CP protein is likely to be a key determinant of the VER outcome in this species, which would be an additional point in common with betanodavirus infection in sea bass.

Although intracranial injection has yielded valuable results, it is an invasive and stressful way of viral inoculation. For this reason, we also performed an RGNNV challenge by immersion, which, in addition to the above‐mentioned advantages, allows pathogens to enter the fish through their natural routes of infection, directly stimulating mucosal immunity via the gills and skin. Although mortality was not observed in RGNNV‐challenged larvae, at least up to 6 dpi, a significant increase of viral genome was recorded at 2 dpi, indicating that the virus could enter and replicate in larvae. However, infection was quickly controlled since viral genome started to decrease at 6 dpi (Figure 5A), just when a slight upregulation of IFN I and apoptosis‐related genes was recorded (Figure 5B,C), with *rtp3* showing the strongest induction, as recorded in the intracranial‐injection assay. Interestingly, no induction of inflammatory genes was recorded in immersion‐challenged larvae, at least until 6 dpi. Thus, as in the previous assays, low or no inflammation seems to be closely related to the control of the infection. Previous results on RGNNV in zebrafish larvae by bath exposure yielded different results. Thus, Morick et al. [40] reported mortality of 4 dpf larvae, whereas no mortality was recorded by Lama et al. [37] with 3 dpf larvae. Differences between viral strains could be responsible for these observations.

Zebrafish larvae have been demonstrated to have important advantages and to be suitable for a variety of approaches. However, some experiments require older individuals; for instance, the evaluation of adaptive responses that are not mature until 4 or 6 weeks post‐fertilization [77] or experimental infections after a feeding trial. For this reason, we also aimed to test the betanodavirus infection model in juvenile zebrafish by intramuscular injection, which is extensively used for experimental NNV infections in fish [48]. The RGNNV genome was detected in zebrafish heads, although viral replication was not recorded (Figure 6A). Thus, the virus migrates to the central nervous system from the injection site, but viral replication does not take place. Interestingly, a quick upregulation of IFN I system (*rtp3* and *irf3*) and apoptosis (*casp8* and *ctsl1a*) genes was recorded at 1 dpi, with *rtp3* being the most induced gene (FC 510) (Figure 6B,C), whereas only a very slight induction of inflammatory‐related genes (*tnfa* and *il1b*) was recorded at 6 dpi. It is well established that the size is a relevant factor regarding susceptibility to betanodaviruses in both natural outbreaks [4, 6, 78] and experimental conditions [49, 60, 78]. Actually, our results agree with those reported by Lama et al. [37], who recorded significant mortality when 3 dpf larvae were intramuscularly infected, whereas no significant mortality was observed when 14 dpf larvae were challenged by the same method.

In summary, zebrafish larvae are susceptible to the three betanodaviruses evaluated, being RGNNV the most virulent isolate. Differences in the virulence of isolates seem to be related with the inflammatory response, which was lower after inoculation with the low‐virulent isolates, RGNNV/SJNNV and SJNNV, in comparison with RGNNV infection. Actually, a low inflammatory response was also recorded after the RGNNV immersion and RGNNV‐injection assays, which did not result in mortality or symptoms, further supporting a relevant role of inflammation on VER outcome. These results support the potential of zebrafish as a model to study betanodavirus–host interaction and prompt further studies to disclose the reasons underlying the observed differences, which could contribute to the demanded development of effective measures to improve farmed fish resistance to viral infections.

## Author Contributions

Conceptualization: Esther García‐Rosado, M. Carmen Alonso, and Julia Béjar. Investigation: Daniel Álvarez‐Torres, Patricia Moreno, Manuel Marí‐Beffa, Manon Mehraz, Maxence Frétaud, Christelle Langevin, and Candela Ravina. Writing – original draft preparation: Daniel Álvarez‐Torres, Patricia Moreno, and Julia Béjar. Writing – review and editing: Daniel Álvarez‐Torres, Patricia Moreno, Esther García‐Rosado, Julia Béjar, Manuel Marí‐Beffa, Maxence Frétaud, Christelle Langevin, and M. Carmen Alonso. Supervision: Esther García‐Rosado, M. Carmen Alonso, Christelle Langevin, and Julia Béjar. Funding acquisition: Esther García‐Rosado, M. Carmen Alonso, Manuel Marí‐Beffa, and Julia Béjar.

## Funding

This study has been financially supported by the projects—Spanish Government (Grant PID2020‐115954RB‐I00/AEI); Next Generation EU Recovery Fund under the Recovery, Transformation and Resilience Plan, European Union, EU, and Regional Government of Andalusia (Grant PCM_000035); the Regional Government (Grant PPRO‐BIO217‐G‐2023); the Regional Government (Grant DGP_PIDI_2024_00807); and Transnational Access Activities From Veterinary Biocontained Research Facility Network Infrastructure From the EU‐H2020 (Grant VBN_22_73). Funding for open‐access publishing: Universidad de Málaga/CBUA.

## Disclosure

All authors have read and agreed to the published version of the manuscript. The opinions expressed are solely those of the authors and do not necessarily reflect those of the EU or the European Commission, EC, the EU and the EC are not responsible of these opinions.

## Conflicts of Interest

The authors declare no conflicts of interest.

## Supporting Information

Additional supporting information can be found online in the Supporting Information section.

## Supporting information


**Supporting Information** Table S1: The list of primers used in this study.

## Data Availability

The data that support the findings of this study are available from the corresponding author upon reasonable request.

## References

[1] Doan Q. K. , Vandeputte M. , Chatain B. , Morin T. , and Allal F. , Viral Encephalopathy and Retinopathy in Aquaculture: A Review, Journal of Fish Diseases. (2017) 40, no. 5, 717–742, 10.1111/jfd.12541. 27633881

[2] Nishizawa T. , Furuhashi M. , Nagai T. , Nakai T. , and Muroga K. , Genomic Classification of Fish Nodaviruses by Molecular Phylogenetic Analysis of the Coat Protein Gene, Applied and Environmental Microbiology. (1997) 63, no. 4, 1633–1636, 10.1128/aem.63.4.1633-1636.1997. PMC168456 9097459

[3] Olveira J. G. , Souto S. , Dopazo C. P. , Thiéry R. , Barja J. L. , and Bandin I. , Comparative Analysis of Both Genomic Segments of Betanodaviruses Isolated From Epizootic Outbreaks in Farmed Fish Species Provides Evidence for Genetic Reassortment, Journal of General Virology. (2009) 90, no. 12, 2940–2951, 10.1099/vir.0.013912-0. 19710256

[4] Toffan A. , Pascoli F. , and Pretto T. , et al.Viral Nervous Necrosis in Gilthead Sea Bream (*Sparus aurata*) Caused by Reassortant Betanodavirus RGNNV/SJNNV: An Emerging Threat for Mediterranean Aquaculture, Scientific Reports. (2017) 7, no. 1, 10.1038/srep46755, 46755. PMC5411978 28462930

[5] Toffolo V. , Negrisolo E. , and Maltese C. , et al.Phylogeny of Betanodaviruses and Molecular Evolution of Their RNA Polymerase and Coat Proteins, Molecular Phylogenetics and Evolution. (2007) 43, no. 1, 298–308, 10.1016/j.ympev.2006.08.003. 16990016

[6] Volpe E. , Gustinelli A. , and Caffara M. , et al.Viral Nervous Necrosis Outbreaks Caused by the RGNNV/SJNNV Reassortant Betanodavirus in Gilthead Sea Bream (*Sparus aurata*) and European Sea Bass (*Dicentrarchus labrax*), Aquaculture. (2020) 523, 10.1016/j.aquaculture.2020.735155, 735155.

[7] Hegde A. , Chen C. L. , Qin Q. W. , Lam T. J. , and Sin Y. M. , Characterization, Pathogenicity and Neutralization Studies of a Nervous Necrosis Virus Isolated From Grouper, *Epinephelus tauvina*, in Singapore, Aquaculture. (2002) 213, no. 1–4, 55–72, 10.1016/S0044-8486(02)00092-3.

[8] Souto S. , Lopez-Jimena B. , Alonso M. D. C. , Garcia-Rosado E. , and Bandin I. , Experimental Susceptibility of European Sea Bass and Senegalese Sole to Different Betanodavirus Isolates, Veterinary Microbiology. (2015) 177, no. 1-2, 53–61, 10.1016/j.vetmic.2015.02.030. 25770892

[9] Carballo C. , Garcia-Rosado E. , Borrego J. J. , and Alonso M. C. , SJNNV Down-Regulates RGNNV Replication in European Sea Bass by the Induction of the Type I Interferon System, Veterinary Research. (2016) 47, no. 1, 10.1186/s13567-015-0304-y, 6. PMC4705746 26743933

[10] Vendramin N. , Toffan A. , and Mancin M. , et al.Comparative Pathogenicity Study of Ten Different Betanodavirus Strains in Experimentally Infected European Sea Bass, *Dicentrarchus labrax* (L.), Journal of Fish Diseases. (2014) 37, no. 4, 371–383, 10.1111/jfd.12117. 23662921

[11] Moreno P. , Lopez-Jimena B. , and Randelli E. , et al.Immuno-Related Gene Transcription and Antibody Response in Nodavirus (RGNNV and SJNNV)-Infected European Sea Bass (*Dicentrarchus labrax* L.), Fish & Shellfish Immunology. (2018) 78, 270–278, 10.1016/j.fsi.2018.04.054. 29702239

[12] García-Álvarez M.Á. , Arizcun M. , Chaves-Pozo E. , and Cuesta A. , Profile of Innate Immunity in Gilthead Seabream Larvae Reflects Mortality Upon Betanodavirus Reassortant Infection and Replication, International Journal of Molecular Sciences. (2022) 23, no. 9, 10.3390/ijms23095092, 5092. PMC9105140 35563482

[13] Trede N. S. , Langenau D. M. , Traver D. , Look A. T. , and Zon L. I. , The Use of Zebrafish to Understand Immunity, Immunity. (2004) 20, no. 4, 367–379, 10.1016/S1074-7613(04)00084-6. 15084267

[14] Stockhammer O. W. , Zakrzewska A. , Hegedûs Z. , Spaink H. P. , and Meijer A. H. , Transcriptome Profiling and Functional Analyses of the Zebrafish Embryonic Innate Immune Response to Salmonella Infection, The Journal of Immunology. (2009) 182, no. 9, 5641–5653, 10.4049/jimmunol.0900082. 19380811

[15] Liu J. , Yuan X. , Fan C. , and Ma G. , Application of the Zebrafish Model in Human Viral Research, Virus Research. (2024) 341, 10.1016/j.virusres.2024.199327, 199327. PMC10835014 38262567

[16] Fajar S. , Dwi S. P. , and Nur I. S. , et al.Zebrafish as a Model Organism for Virus Disease Research: Current Status and Future Directions, Heliyon. (2024) 10, no. 13, 10.1016/j.heliyon.2024.e33865. PMC11282986 39071624

[17] Ludwig M. , Palha N. , and Torhy C. , et al.Whole-Body Analysis of a Viral Infection: Vascular Endothelium is a Primary Target of Infectious Hematopoietic Necrosis Virus in Zebrafish Larvae, PLoS Pathogens. (2011) 7, no. 2, 10.1371/journal.ppat.1001269. PMC3033377 21304884

[18] Briolat V. , Jouneau L. , and Carvalho R. , et al.Contrasted Innate Responses to Two Viruses in Zebrafish: Insights Into the Ancestral Repertoire of Vertebrate IFN-Stimulated Genes, The Journal of Immunology. (2014) 192, no. 9, 4328–4341, 10.4049/jimmunol.1302611. 24683187

[19] Marana M. H. , Schmidt J. G. , Biacchesi S. , Lorenzen N. , and Jørgensen L. V. G. , Zebrafish (*Danio rerio*) Larvae as a Model for Real-Time Studies of Propagating VHS Virus Infection, Tissue Tropism and Neutrophil Activity, Journal of Fish Diseases. (2021) 44, no. 5, 563–571, 10.1111/jfd.13294. 33170959

[20] LaPatra S. E. , Barone L. , Jones G. R. , and Zon L. I. , Effects of Infectious Hematopoietic Necrosis Virus and Infectious Pancreatic Necrosis Virus Infection on Hematopoietic Precursors of the Zebrafish, Blood Cells, Molecules, and Diseases. (2000) 26, no. 5, 445–452, 10.1006/bcmd.2000.0320. 11112382

[21] Phelan P. , Pressley M. , Witten P. , Mellon M. , Blake S. , and Kim C. H. , Characterization of Snakehead Rhabdovirus Infection in Zebrafish (*Danio rerio*), Journal of Virology. (2005) 79, no. 3, 1842–1852, 10.1128/JVI.79.3.1842-1852.2005. PMC544118 15650208

[22] Wang Z. L. , Xu X. P. , and He B. L. , et al.Infectious Spleen and Kidney Necrosis Virus OFR48R Functions as a New Viral Vascular Endothelial Growth Factor, Journal of Virology. (2008) 82, no. 9, 4371–4383, 10.1128/JVI.02027-07. PMC2293046 18305039

[23] Xu X. , Zhang L. , and Weng S. , et al.A Zebrafish (*Danio rerio*) Model of Infectious Spleen and Kidney Necrosis Virus (ISKNV) Infection, Virology. (2008) 376, no. 1, 1–12, 10.1016/j.virol.2007.12.026. 18423508

[24] Martín V. , Mavian C. , and López Bueno A. , et al.Establishment of a Zebrafish Infection Model for the Study of Wild-Type and Recombinant European Sheatfish Virus, Journal of Virology. (2015) 89, no. 20, 10702–10706, 10.1128/JVI.01580-15. PMC4580169 26246565

[25] Sanders G. E. , Batts W. N. , and Winton J. R. , Susceptibility of Zebrafish (*Danio rerio*) to a Model Pathogen, Spring Viremia of Carp Virus, Comparative Medicine. (2003) 53, no. 5, 514–521. 14655994

[26] Wang Y. , Zhang H. , and Lu Y. , et al.Comparative Transcriptome Analysis of Zebrafish (*Danio rerio*) Brain and Spleen Infected With Spring Viremia of Carp Virus (SVCV), Fish & Shellfish Immunology. (2017) 69, 35–45, 10.1016/j.fsi.2017.07.055. 28757199

[27] Medina-Gali R. M. , del Mar Ortega-Villaizan M. , Mercado L. , Novoa B. , Coll J. , and Perez L. , Beta-Glucan Enhances the Response to SVCV Infection in Zebrafish, Developmental & Comparative Immunology. (2018) 84, 307–314, 10.1016/j.dci.2018.02.019. 29524446

[28] Medina-Gali R. , Bello-Perez M. , and Ciordia S. , et al.Plasma Proteomic Analysis of Zebrafish Following Spring Viremia of Carp Virus Infection, Fish & Shellfish Immunology. (2019) 86, 892–899, 10.1016/j.fsi.2018.12.035. 30580041

[29] Liu R. , Hu X. , and Lü A. , et al.Proteomic Profiling of Zebrafish Challenged by Spring Viremia of Carp Virus Provides Insight Into Skin Antiviral Response, Zebrafish. (2020) 17, no. 2, 91–103, 10.1089/zeb.2019.1843. 32176570

[30] Levraud J.-P. , Palha N. , Langevin C. , and Boudinot P. , Through the Looking Glass: Witnessing Host–Virus Interplay in Zebrafish, Trends in Microbiology. (2014) 22, no. 9, 490–497, 10.1016/j.tim.2014.04.014. 24865811

[31] Lieschke G. J. and Currie P. D. , Animal Models of Human Disease: Zebrafish Swim Into View, Nature Reviews Genetics. (2007) 8, no. 5, 353–367, 10.1038/nrg2091. 17440532

[32] Willett C. E. , Cortes A. , Zuasti A. , and Zapata A. G. , Early Hematopoiesis and Developing Lymphoid Organs in the Zebrafish, Developmental Dynamics. (1999) 214, no. 4, 323–336. 10.1002/(SICI)1097-0177(199904)214:4<323::AID-AJA5>3.0.CO;2-3 10213388

[33] Truong L. , Bieberich A. A. , Fatig R. O.III, Rajwa B. , Simonich M. T. , and Tanguay R. L. , Accelerating Antiviral Drug Discovery: Early Hazard Detection With a Dual Zebrafish and Cell Culture Screen of a 403 Compound Library, Archives of Toxicology. (2025) 99, no. 3, 1029–1041, 10.1007/s00204-024-03948-3. PMC11821682 39730949

[34] Pereiro P. , Figueras A. , and Novoa B. , Compilation of Antiviral Treatments and Strategies to Fight Fish Viruses, Reviews in Aquaculture. (2021) 13, no. 3, 1223–1254, 10.1111/raq.12521.

[35] Hernández-Urcera J. , Romero A. , and Cruz P. , et al.Screening of Microalgae for Bioactivity With Antiviral, Antibacterial, Anti-Inflammatory and Anti-Cancer Assays, Biology. (2024) 13, no. 4, 10.3390/biology13040255, 255. PMC11048355 38666867

[36] Lu M. W. , Chao Y. M. , and Guo T. C. , et al.The Interferon Response is Involved in Nervous Necrosis Virus Acute and Persistent Infection in Zebrafish Infection Model, Molecular Immunology. (2008) 45, no. 4, 1146–1152, 10.1016/j.molimm.2007.07.018. 17727953

[37] Lama R. , Pereiro P. , Figueras A. , and Novoa B. , Zebrafish as a Vertebrate Model for Studying *Nodavirus* Infections, Frontiers in Immunology. (2022) 13, 10.3389/fimmu.2022.863096, 863096. PMC8987509 35401537

[38] Morick D. and Saragovi A. , Inhibition of Nervous Necrosis Virus by Ribavirin in a Zebrafish Larvae Model, Fish & Shellfish Immunology. (2017) 60, 537–544, 10.1016/j.fsi.2016.11.015. 27833011

[39] Binesh C. P. , Mortality Due to Viral Nervous Necrosis in Zebrafish *Danio rerio* and Goldfish *Carassius auratus* , Diseases of Aquatic Organisms. (2013) 104, no. 3, 257–260, 10.3354/dao02605. 23759563

[40] Morick D. , Faigenbaum O. , Smirnov M. , Fellig Y. , Inbal A. , and Kotler M. , Mortality Caused by Bath Exposure of Zebrafish (*Danio rerio*) Larvae to Nervous Necrosis Virus is Limited to the Fourth Day Postfertilization, Applied and Environmental Microbiology. (2015) 81, no. 10, 3280–3287, 10.1128/AEM.04175-14. PMC4407219 25746990

[41] Iwamoto T. , Nakai T. , Mori K. , Arimoto M. , and Furusawa I. , Cloning of the Fish Cell Line SSN-1 for Piscine Nodaviruses, Diseases of Aquatic Organisms. (2000) 43, 81–89, 10.3354/dao043081. 11145456

[42] Moreno P. , Souto S. , Leiva-Rebollo R. , Borrego J. J. , Bandín I. , and Alonso M. C. , Capsid Amino Acids at Positions 247 and 270 are Involved in the Virulence of Betanodaviruses to European Sea Bass, Scientific Reports. (2019) 9, no. 1, 10.1038/s41598-019-50622-1, 14068. PMC6773868 31575937

[43] Reed L. J. and Müench H. , A Simple Method of Estimating 50% End Points, American Journal of Epidemiology. (1938) 27, no. 3, 493–497, 10.1093/oxfordjournals.aje.a118408.

[44] Toffan A. , Panzarin V. , Toson M. , Cecchettin K. , and Pascoli F. , Water Temperature Affects Pathogenicity of Different Betanodavirus Genotypes in Experimentally Challenged European Sea Bass *Dicentrarchus labrax* , Diseases of Aquatic Organisms. (2016) 119, no. 3, 231–238, 10.3354/dao03003. 27225206

[45] Moreno P. , Garcia-Rosado E. , Borrego J. J. , and Alonso M. C. , Genetic Characterization and Transcription Analyses of the European Sea Bass (*Dicentrarchus labrax*) *Isg15* Gene, Fish & Shellfish Immunology. (2016) 55, 642–646, 10.1016/j.fsi.2016.06.043. 27368533

[46] Renshaw S. A. , Loynes C. A. , Trushell D. M. , Elworthy S. , Ingham P. W. , and Whyte M. K. , A Transgenic Zebrafish Model of Neutrophilic Inflammation, Blood. (2006) 108, no. 13, 3976–3978, 10.1182/blood-2006-05-024075. 16926288

[47] de Oliveira S. , Reyes-Aldasoro C. C. , Candel S. , Renshaw S. A. , Mulero V. , and Calado Â. , Cxcl8 (IL-8) Mediates Neutrophil Recruitment and Behavior in the Zebrafish Inflammatory Response, The Journal of Immunology. (2013) 190, no. 8, 4349–4359, 10.4049/jimmunol.1203266. PMC3736093 23509368

[48] Peducasse S. , Castric J. , Thiery R. , Jeffroy J. , Le Ven A. , and Laurencin F. B. , Comparative Study of Viral Encephalopathy and Retinopathy in Juvenile Sea Bass *Dicentrarchus labrax* Infected in Different Ways, Diseases of Aquatic Organisms. (1999) 36, no. 1, 11–20, 10.3354/dao036011. 10349548

[49] Bandín I. and Souto S. , Betanodavirus and VER Disease: A 30-Year Research Review, Pathogens. (2020) 9, no. 2, 10.3390/pathogens9020106, 106. PMC7168202 32050492

[50] Padrós F. , Caggiano M. , Toffan A. , Constenla M. , Zarza C. , and Ciulli S. , Integrated Management Strategies for Viral Nervous Necrosis (VNN) Disease Control in Marine Fish Farming in the Mediterranean, Pathogens. (2022) 11, no. 3, 10.3390/pathogens11030330, 330. PMC8955002 35335654

[51] Achenbach J. C. , Leggiadro C. , Sperker S. A. , Woodland C. , and Ellis L. D. , Comparison of the Zebrafish Embryo Toxicity Assay and the General and Behavioral Embryo Toxicity Assay as New Approach Methods for Chemical Screening, Toxics. (2020) 8, no. 4, 10.3390/toxics8040126, 126. PMC7767334 33371320

[52] Maerz L. D. , Casar Tena T. , Gerhards J. , Donow C. , Jeggo P. A. , and Philipp M. , Analysis of Cilia Dysfunction Phenotypes in Zebrafish Embryos Depleted of Origin Recognition Complex Factors, European Journal of Human Genetics. (2019) 27, no. 5, 772–782, 10.1038/s41431-019-0338-0. PMC6461852 30696958

[53] Andrades J. A. , Becerra J. , and Fernández-Llebrez P. , Skeletal Deformities of the Gilthead Sea Bream (*Sparus aurata*, L.): Study of the Subcommissural Organ (SCO) and Reissner’s Fiber (RF), Annals of Anatomy - Anatomischer Anzeiger. (1994) 176, no. 4, 381–383, 10.1016/S0940-9602(11)80524-8. 8085662

[54] Printzi A. , Mazurais D. , and Witten P. E. , et al.Juvenile Zebrafish (*Danio rerio*) are Able to Recover From Lordosis, Scientific Reports. (2022) 12, no. 1, 10.1038/s41598-022-26112-2, 21533. PMC9748118 36513797

[55] Muñoz-Montecinos C. , Romero A. , and Sepúlveda V. , et al.Turning the Curve Into Straight: Phenogenetics of the Spine Morphology and Coordinate Maintenance in the Zebrafish, Frontiers in Cell and Developmental Biology. (2022) 9, 10.3389/fcell.2021.801652, 801652. PMC8826430 35155449

[56] Kim J. O. , Kim J. O. , Kim W. S. , and Oh M. J. , Characterization of the Transcriptome and Gene Expression of Brain Tissue in Sevenband Grouper (*Hyporthodus septemfasciatus*) in Response to NNV Infection, Genes. (2017) 8, no. 1, 10.3390/genes8010031, 31. PMC5295026 28098800

[57] Labella A. M. , Garcia-Rosado E. , and Bandín I. , et al.Transcriptomic Profiles of Senegalese Sole Infected With Nervous Necrosis Virus Reassortants Presenting Different Degree of Virulence, Frontiers in Immunology. (2018) 9, 10.3389/fimmu.2018.01626, 1626. PMC6056728 30065724

[58] Moreno P. , Gemez-Mata J. , and Garcia-Rosado E. , et al.Differential Immunogene Expression Profile of European Sea Bass (*Dicentrarchus labrax*, L.) in Response to Highly and Low Virulent NNV, Fish & Shellfish Immunology. (2020) 106, 56–70, 10.1016/j.fsi.2020.06.052. 32702480

[59] Chattopadhyay S. , Marques J. T. , and Yamashita M. , et al.Viral Apoptosis is Induced by IRF-3-Mediated Activation of Bax, The EMBO Journal. (2010) 29, no. 10, 1762–1773, 10.1038/emboj.2010.50. PMC2876960 20360684

[60] Gemez-Mata J. , Moreno P. , Alvarez-Torres D. , Garcia-Rosado E. , Bejar J. , and Alonso M. C. , Comparative Analysis of Immune Gene Transcription in Sea Bream (*Sparus aurata*) Challenged With RGNNV or RGNNV/SJNNV Betanodaviruses, Pathogens. (2024) 13, no. 6, 10.3390/pathogens13060478, 478. PMC11207047 38921776

[61] Gémez-Mata J. , Labella A. M. , Bandín I. , Borrego J. J. , and García-Rosado E. , Immunogene Expression Analysis in Betanodavirus Infected-Senegalese Sole Using an OpenArray® Platform, Gene. (2021) 774, 10.1016/j.gene.2021.145430, 145430. 33444680

[62] Liu P. , Wang L. , Kwang J. , Yue G. H. , and Wong S. M. , Transcriptome Analysis of Genes Responding to NNV Infection in Asian Seabass Epithelial Cells, Fish & Shellfish Immunology. (2016) 54, 342–352, 10.1016/j.fsi.2016.04.029. 27109582

[63] Moreno P. , Alvarez-Torres D. , Garcia-Rosado E. , Bejar J. , and Alonso M. C. , Functional Assessment of European Sea Bass RTP3 Proteins and Their Effect on RGNNV Replication, Fish & Shellfish Immunology. (2026) 168, 10.1016/j.fsi.2025.111002, 111002. 41265802

[64] Liu P. , Wang L. , Ye B. Q. , Huang S. , Wong S. M. , and Yue G. H. , Characterization of a Novel Disease Resistance Gene *rtp3* and Its Association With VNN Disease Resistance in Asian Seabass, Fish & Shellfish Immunology. (2017) 61, 61–67, 10.1016/j.fsi.2016.12.021. 27989862

[65] Novoa B. , Mackenzie S. , and Figueras A. , Inflammation and Innate Immune Response Against Viral Infections in Marine Fish, Current Pharmaceutical Design. (2010) 16, no. 38, 4175–4184, 10.2174/138161210794519156. 21184664

[66] Robertson T. F. and Huttenlocher A. , Real-Time Imaging of Inflammation and Its Resolution: It’s Apparent because It’s Transparent, Immunological Reviews. (2022) 306, no. 1, 258–270, 10.1111/imr.13061. PMC8855992 35023170

[67] Rose C. D. , Pompili D. , and Henke K. , et al.SCO-Spondin Defects and Neuroinflammation Are Conserved Mechanisms Driving Spinal Deformity Across Genetic Models of Idiopathic Scoliosis, Current Biology. (2020) 30, no. 12, 2363–2373.e6, 10.1016/j.cub.2020.04.020. 32386528

[68] Overgard A. C. , Nerland A. H. , Fiksdal I. U. , and Patel S. , Atlantic Halibut Experimentally Infected With Nodavirus Shows Increased Levels of -Cells Markers and IFN Transcripts, Developmental & Comparative Immunology. (2012) 37, no. 1, 139–150, 10.1016/j.dci.2011.10.003. 22020051

[69] Poisa-Beiro L. , Dios S. , Montes A. , Aranguren R. , Figueras A. , and Novoa B. , Nodavirus Increases the Expression of Mx and Inflammatory Cytokines in Fish Brain, Molecular Immunology. (2008) 45, no. 1, 218–225, 10.1016/j.molimm.2007.04.016. 17543386

[70] Pereiro P. , Figueras A. , and Novoa B. , RNAseq Analysis of Juvenile Gilthead Sea Bream (*Sparus aurata*) Provides Some Clues Regarding Their Resistance to the Nodavirus RGNNV Genotype, Fish & Shellfish Immunology. (2023) 134, 10.1016/j.fsi.2023.108588, 108588. 36740080

[71] Sullivan C. , Soos B.-L. , Millard P. J. , Kim C. H. , and King B. L. , Modeling Virus-Induced Inflammation in Zebrafish: A Balance Between Infection Control and Excessive Inflammation, Frontiers in Immunology. (2021) 12, 10.3389/fimmu.2021.636623, 636623. PMC8138431 34025644

[72] Costa J. Z. and Thompson K. D. , Understanding the Interaction Between Betanodavirus and Its Host for the Development of Prophylactic Measures for Viral Encephalopathy and Retinopathy, Fish & Shellfish Immunology. (2016) 53, 35–49, 10.1016/j.fsi.2016.03.033. 26997200

[73] Su Y. C. , Wu J. L. , and Hong J. R. , *Betanodavirus* Non-Structural Protein B2: A Novel Necrotic Death Factor that Induces Mitochondria-Mediated Cell Death in Fish Cells, Virology. (2009) 385, no. 1, 143–154, 10.1016/j.virol.2008.11.036. 19116179

[74] Su Y. C. , Chiu H. W. , Hung J. C. , and Hong J. R. , *Beta-nodavirus*, B2 Protein Induces Hydrogen Peroxide Production, Leading to *Drp1*-Recruited Mitochondrial Fragmentation and Cell Death via Mitochondrial Targeting, Apoptosis. (2014) 19, no. 10, 1457–1470, 10.1007/s10495-014-1016-x. PMC4167032 25008790

[75] Gémez-Mata J. , Álvarez-Torres D. , García-Rosado E. , Alonso M. C. , and Béjar J. , Comparative Analysis of Marine and Freshwater Viral Haemorrhagic Septicaemia Virus (VHSV) Isolates Antagonistic Activity, Comparative Immunology, Microbiology and Infectious Diseases. (2020) 69, 10.1016/j.cimid.2020.101426, 101426. 32014622

[76] Souto S. , Olveira J. G. , Dopazo C. P. , and Bandín I. , Reassortant Betanodavirus Infection in Turbot (*Scophthalmus maximus*), Journal of Fish Diseases. (2016) 39, no. 11, 1347–1356, 10.1111/jfd.12466. 27135777

[77] Lam S. H. , Chua H. L. , Gong Z. , Lam T. J. , and Sin Y. M. , Development and Maturation of the Immune System in Zebrafish, *Danio rerio*: A Gene Expression Profiling, *In Situ* Hybridization and Immunological Study, Developmental & Comparative Immunology. (2004) 28, no. 1, 9–28, 10.1016/S0145-305X(03)00103-4. 12962979

[78] Toffan A. , Biasini L. , and Pretto T. , et al.Age Dependency of RGNNV/SJNNV Viral Encephalo-Retinopathy in Gilthead Sea Bream (*Sparus aurata*), Aquaculture. (2021) 539, 10.1016/j.aquaculture.2021.736605, 736605.

