# Supplementary material for: Zebrafish Reveals Genotype‐Specific Differences in Betanodavirus Infection and Host Responses
Source: Transbound Emerg Dis. 2026 Oct 30;2026:8111758. doi: 10.1155/tbed/8111758 (PMC13720760; doi:10.1155/tbed/8111758)
Supplement: Supplementary file 1 [file TBED-2026-8111758-s001.docx]

**Table 1**. Primers used in this study.

| Target gene | Code | Nucleotide sequence (5´-3´) | Reference |
| --- | --- | --- | --- |
| Housekeeping gene | | | |
| *Actin beta 1* | *actb1* | F: ATGGATGAGGAAATCGCTG | 40 |
|  |  | R: ATGCCAACCATCACTCCCT |  |
| IFN-I system genes | | | |
| Myxovirus (influenza) resistance A | *mxa* | F: GACCGTCTCTGATGTGGTTA | 82 |
|  |  | R: GCATGCTTTAGACTCTGGCT |  |
| ISG15 ubiquitin like modifier | *isg15* | F: TAATGCCACAGTCGGTGAA | 79 |
|  |  | R: AGGTCCAGTGTTAGTGATGAGC |  |
| *Receptor transporter protein 3* | *rtp3* | F: ttgcactcctgtttgaagcc | This study |
|  |  | R: catctctggctctttcgcac |  |
| *Interferon regulatory factor 3* | *irf3* | F: CAAAACCGCTGTTCGTGCC  R: CATCGTCGCTGTTGGAGTCCT | 82 |
| Apoptosis genes | | | |
| *Caspase 8* | *casp8* | F: CCAGACAATCTGGATGAACTTTAC | 80 |
|  |  | R: TGCAAACTGCTTTATCTCATCT |  |
| *Caspase 9* | *casp9* | F: CTGAGGCAAGCCATAATCG | 80 |
|  |  | R: AGAGGACATGGGAATAGCGT |  |
| *Cathepsin La* | *ctsl1a* | F: acgaggagttcagacaggtg | This study |
|  |  | R: tcccttctctctccagtcca |  |
| Inflammatory response genes | | | |
| *Tumour necrosis factor a* | *tnfa* | F: GCGCTTTTCTGAATCCTACG | 40 |
|  |  | R: TGCCCAGTCTGTCTCCTTCT |  |
| *Interleukin 1 beta* | *il1b* | F: GAACAGAATGAAGCACATCAAACC | 82 |
|  |  | R: ACGGCACTGAATCCACCAC |  |
| *Interleukin 6* | *il6* | F: GCCAACTGCAACATACCAAA | 37 |
|  |  | R: ACTGACAGCACGCAAAACTC |  |
| *Interleukin 8* | *il8* | F: GTCGCTGCATTGAAACAGAA | 83 |
|  |  | R: CTTAACCCATGGAGCAGAGG |  |
| Viral genes | | | |
| *RGNNV RNA2* | *RG-RNA2* | F: ACCGTCCGCTGTCTATTGACTA | 45 |
|  |  | R: CAGATGCCCCAGCGAAACC |  |
| *RG/SJ and SJNNV RNA2* | *SJ-RNA2* | F: GACACCACCGCTCCAATTACTAC | 81 |
|  |  | R: ACGAAATCCAGTGTAACCGTTGT |  |

79. B.Chen, C. Li, J. Yao, et al., “Zebrafish NIK Mediates IFN Induction by Regulating Activation of IRF3 and NF-κB,” *The Journal of Immunology*, vol. 204, no 7, pp. 1881-1891, 2020.

80. Z. L. Jia, J. Cen, J. B. Wang, et al., “Mechanism of Isoniazid-Induced Hepatotoxicity in Zebrafish Larvae: Activation of ROS-Mediated ERS, Apoptosis and the Nrf2 Pathway,” *Chemosphere*, vol. 227, pp. 541-550, 2019.

81. B. Lopez‐Jimena, E. Garcia‐Rosado, C. Infante, D. Castro, J. J. Borrego, and M. Carmen Alonso, “Effect of the Coexistence on the Replication of Striped Jack Nervous Necrosis Virus (SJNNV) and Red‐Spotted Grouper Nervous Necrosis Virus (RGNNV) Using an *In Vitro* Approach,” *Journal of Applied Ichthyology*, vol. 30, no 5, pp. 916-922, 2014.

82. K., Rakus, M. Mojzesz, M. Widziolek, et al., “Antiviral Response of Adult Zebrafish (*Danio rerio*) During Tilapia Lake Virus (TiLV) Infection, *Fish & Shellfish Immunology*, vol. 101, pp. 1-8, 2020.

83. L. Song, X. Zhu, Y. Zhou, et al., “Establishment of a Rotavirus-Infected Zebrafish Model and Its Application in Drug Screening,” *Biomedicine & Pharmacotherapy*, vol. 145, Article ID 112398, 2022.
